# Applying phasor approach analysis of multiphoton FLIM measurements to probe the metabolic activity of three-dimensional *in vitro* cell culture models

**DOI:** 10.1038/srep42730

**Published:** 2017-02-13

**Authors:** Pirmin H. Lakner, Michael G. Monaghan, Yvonne Möller, Monilola A. Olayioye, Katja Schenke-Layland

**Affiliations:** 1 Department of Women’s Health, Research Institute for Women’s Health, University Hospital of the Eberhard Karls University Tübingen, Tübingen, Germany; 2 Trinity Centre for Bioengineering, Trinity Biomedical Sciences Institute, Trinity College Dublin, Dublin, Ireland; 3 Department of Mechanical and Manufacturing Engineering, School of Engineering, Trinity College Dublin, Dublin, Ireland; 4 Department of Cell and Tissue Engineering, Fraunhofer Institute for Interfacial Engineering and Biotechnology (IGB), Stuttgart, Germany; 5 Institute of Cell Biology and Immunology, University of Stuttgart, Stuttgart, Germany; 6 Center for Personalised Medicine (ZPM), University Hospital of the Eberhard Karls University Tübingen, Tübingen, Germany; 7 Stuttgart Research Center Systems Biology, University of Stuttgart, Stuttgart, Germany; 8 Department of Medicine/Cardiology, University of California Los Angeles (UCLA), Los Angeles/CA, USA

## Abstract

Fluorescence lifetime imaging microscopy (FLIM) can measure and discriminate endogenous fluorophores present in biological samples. This study seeks to identify FLIM as a suitable method to non-invasively detect a shift in cellular metabolic activity towards glycolysis or oxidative phosphorylation in 3D Caco-2 models of colorectal carcinoma. These models were treated with potassium cyanide or hydrogen peroxide as controls, and epidermal growth factor (EGF) as a physiologically-relevant influencer of cell metabolic behaviour. Autofluorescence, attributed to nicotinamide adenine dinucleotide (NADH), was induced by two-photon laser excitation and its lifetime decay was analysed using a standard multi-exponential decay approach and also a novel custom-written code for phasor-based analysis. While both methods enabled detection of a statistically significant shift of metabolic activity towards glycolysis using potassium cyanide, and oxidative phosphorylation using hydrogen peroxide, employing the phasor approach required fewer initial assumptions to quantify the lifetimes of contributing fluorophores. 3D Caco-2 models treated with EGF had increased glucose consumption, production of lactate, and presence of ATP. FLIM analyses of these cultures revealed a significant shift in the contribution of protein-bound NADH towards free NADH, indicating increased glycolysis-mediated metabolic activity. This data demonstrate that FLIM is suitable to interpret metabolic changes in 3D *in vitro* models.

Fluorescence lifetime imaging microscopy (FLIM) is a useful imaging tool that can be utilized for the non-invasive characterisation of biological samples *in vitro*^1^ and *in vivo*^2^, and serve as a method for *in situ* diagnosis of pathological tissues^3^. The nature of multiphoton imaging allows it to achieve spatial resolutions at a nanometre and picosecond time scale^2^,^4^.

Anabolic reactions in eukaryotic and prokaryotic cells require nicotinamide adenine dinucleotide (NADH), which is predominantly active in the mitochondria^5^. Chance *et al*. demonstrated that this important cofactor exhibits autofluorescence when excited at particular wavelengths^6^, making it suitable as an endogenous fluorophore for non-invasive imaging using autofluorescence microscopy^7^. As NADH and reduced nicotinamide adenine dinucleotide phosphate (NADPH) possess similar excitation and emission wavelengths, and equal fluorescence lifetimes (in solution)^8^, both signals are acquired simultaneously using FLIM. In biological samples, NADPH has a concentration that is five times lower than NADH^9^ and a quantum yield that is 1.25–2.5 times lower^10^, and therefore, NADH is more commonly considered in FLIM. NADH can occur in a free or protein-bound state in cells, which is discriminated due to the fluorescence decay time of the emission signal that is measurable using FLIM. Free NADH has a short fluorescence lifetime τ_1_ (0.3–0.8 ns), and protein-bound NADH has a long fluorescence lifetime τ_2_ (1–6.5 ns)^11^.

Standard mathematical methods applied to FLIM data analyse emitted photon intensities, photon decay times (lifetimes) and their contributions. A change in the distribution of the protein-bound and unbound forms of NADH can be interpreted as a change in the balance between glycolysis and oxidative phosphorylation^12^. For time domain FLIM measurements with time correlated single photon counting (TCSPC), lifetimes τ_n_ and their contributions α_n_ are modelled and calculated using multi-exponential decay fittings (MEDF)^13^. The number of exponential components *n* is chosen so the chi-squared goodness of fit (χ^2^) is as close as possible to unity:


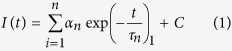


where I(t) is the intensity of photons at time *t* and *C* is offset (e.g. background noise, room light, scattering effects), and the contributing lifetimes and their distributions are estimated using multiple iterations (please see the Supplementary data file for a more detailed explanation). Another approach to calculate fluorescence lifetimes is the phasor approach^14^,^15^. An exponential decay curve can be mathematically modelled using the following equation:


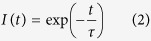


and in the case of a periodic signal (e.g. excitation using a pulsed laser signal) with repetition frequency ω, such as is the case with TCSPC, the lifetimes can be transformed using Fourier transform:


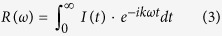


The result is a complex number, which, for further calculations, is separated in the real and complex part:


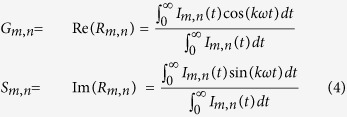


where Re and Im are the real and complex components of the Fourier transform respectively, component I(t) is the photon intensity, and m and n are indices for row and column of input images (please see the Supplementary data file for a more detailed explanation).

By plotting the imaginary versus real component of the transform, lifetimes of emitted signals within pixels of an image obtained using FLIM, are assigned to a point in a phasor plot. For mono-exponential decay, all points lay on a semicircle beginning in (1.0) moving counter clockwise to (0.0) on a semicircle with radius of 0.5^16^,^17^, which is called the universal circle. Towards the linear fitting of phasor approach data points, the total least square (TLS) method is applied to account for errors in both spatial dimensions (G and S)^18^. By calculating the intersections of the fitted linear function with the universal circle, the contribution lifetimes (τ_1_ and τ_2_) can be calculated:


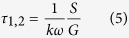


The principle advantage of a phasor approach when compared with MEDF is that it requires less initial assumptions of the components contributing to fluorescence decay profiles and requires no iterative calculations^19^.

Biomedical researchers are increasingly designing three-dimensional (3D) *in vitro* models that can more accurately mimic human healthy or pathological tissues in order to study complex developmental processes, develop new drugs, reduce the need for pre-clinical animal testing, or even generate patient-tailored *in vitro* models of various diseases^20^. Such applications have been widely tested in applications of skin^21^, colorectal cancer^22^, breast cancer^22^, and cardiac applications^23^. Assessment of such 3D *in vitro* models is often destructive due to the need for histological, histochemical or immunohistological staining as well as the elucidation of molecular pathways via gene expression analyses. Non-invasive interpretation of such models is garnering interest but can be limited to monolayer cultures or macroscale analysis^24^. The nature of multiphoton imaging enables the penetration of tissues, biopsies and such *in vitro* models, enabling 3D imaging^25^.

This study aims to employ the feature of multiphoton laser-induced depth penetration to discern changes in metabolic activity in 3D *in vitro* models using the human Caco-2 colorectal adenocarcinoma cell line. The Caco-2 cell line is an adenomacarcinoma cell line that forms tight junctions and is thus commonly used to study barrier function in 2D monolayers^26^. In 3D matrix-containing cultures, Caco-2 cells initially proliferate to form fully polarized, growth-arrested cysts with a central hollow lumen, recapitulating important features of the intestinal epithelium found *in vivo*^27^. In advanced stages of cancer, cells are characterized by hyperproliferation and the loss of polarity^28^. 3D Caco-2 cultures are thus an ideal model to study different stages of transformation, including escape from growth arrest, polarity loss and matrix invasion. Stimulation of 3D Caco-2 cultures with the growth factor EGF increases cell proliferation leading to larger cysts that maintain their polarity, thus mimicking an early stage of cancer progression^29^. This increase in proliferation is expected to be associated with measurable changes in metabolic activity. Metabolic changes are often used as surrogate markers to analyse tumour development and growth or response to drug treatment. As the analysis of metabolic profiles typically requires labelling-agents or immunohistological staining of fixed cultures, we sought to develop a label-free technique that could possibly be applied towards Caco-2 3D cultures as an *in vitro* model of colorectal cancer. Here, we used a standard multi-exponential decay approach to interpret FLIM data obtained from 3D colorectal cancer models and compared the data with interpretations from a novel custom-designed phasor plot approach based on Fourier transform. In addition, chemical modifiers of metabolism such as potassium cyanide (KCN), to inhibit oxidative phosphorylation, and hydrogen peroxide (H_2_O_2_), to increase oxidative phosphorylation in 3D Caco-2 luminal cyst models, were used to validate the detected differences using both a standard multi-exponential decay approach and the phasor plot approach. We also treated the 3D *in vitro* models with EGF, which binds to the ErbB1 receptor (also known as EGFR or HER1), leading to multiple pathway activations that lead, among other things, to an increased DNA synthesis and cellular proliferation^30^.

## Results

### Induced metabolic shifts can be detected in 3D *in vitro* models of colorectal cancer using FLIM and multi-exponential decay analyses

**Figure 1:**
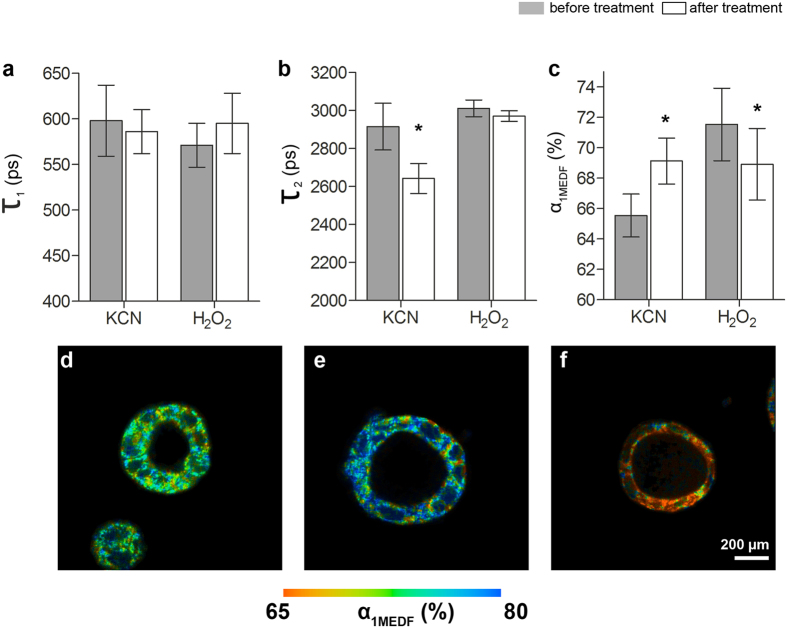
Multi-exponential decay fitting FLIM characteristics of 3D Caco-2 luminal cysts which are untreated and treated with KCN or H_2_O_2_. (**a**) *τ*_1_ of 3D Caco-2 luminal cysts before and after treatment with KCN (n = 6) or H_2_O_2_ (n = 4). No significant impact is seen on the short fluorescence lifetime *τ*_1_of NADH. (**b**)*τ*_2_ of 3D Caco-2 luminal cysts before and after treatment with KCN or H_2_O_2_. Treatment with KCN induces a significant decrease of *τ*_2_ (n = 6, *p = 0.0004), whereas no significant impact was detected on *τ*_2_ when treated with H_2_O_2_. (**c**) Changes are seen in the short fluorescence lifetime contribution *α*_1MEDF_ before and after treating 3D Caco-2 luminal cysts with KCN or H_2_O_2_: Treatment with KCN induces an increase in *α*_1MEDF_ (n = 6, *p = 0.0029), whereas H_2_O_2_ treatment leads to a decrease in *α*_1MEDF_ (n = 4, *p = 0.0434). (**d–f**) False color-coded images of (**d)** untreated, (**e)** KCN-treated, and (**f**) H_2_O_2_-treated 3D Caco-2 luminal cyst models indicating the range of *α*_1MEDF_ from 65% (red) to 80% (blue).

FLIM measurements were performed utilizing 3D Caco-2 luminal cysts. A multi-exponential decay fitting was applied to the acquired FLIM data using SPCImage software (v5.4.0, Becker & Hickl GmbH, Berlin, Germany). An instrument response function (IRF) data file was recorded from urea crystals and applied to minimize the chi-squared fit χ^2^, which is an indicator of how the measured data and fitted equations are related. χ^2^ = 1 represents complete unity between the measured data and theoretical fit. 3D Caco-2 luminal cysts were imaged using FLIM before and after the application of KCN or H_2_O_2_. A bi-exponential decay fitting was applied to the fluorescent decay profiles of the 3D *in vitro* models. From this fitting, the individual contributing lifetime components of free NADH (τ_1_), protein-bound NADH (τ_2_) and the contribution of their lifetimes to the overall signal (α_1MEDF_ being the contribution of τ_1_, Fig. 1) were assessed. No significant difference was detected before or after the treatment of KCN or H_2_O_2_ with regard to τ_1_ (Fig. 1a). However, treating 3D Caco-2 models with KCN yielded in a significant reduction in the lifetime of τ_2_ (2915 ± 123 ps versus 2642 ± 79 ps, n = 6, p = 0.0004, Fig. 1b). Most remarkably, KCN and H_2_O_2_ were capable of eliciting statistically significant changes in the contribution of the lifetimes τ_1_ and τ_2_ (Fig. 1c). KCN-treated 3D Caco-2 luminal cysts had a significantly higher α_1MEDF_ (65.65 ± 1.41% versus 69.13 ± 1.51%, n = 6, p = 0.0029, Fig. 1c), and H_2_O_2_ caused a statistically significant decrease in α_1MEDF_ (71.53 ± 2.39% versus 68.91 ± 2.36%, n = 4, p = 0.0434). The results from the multi-exponential decay fitting can be viewed qualitatively in the false colour-coded micrographs provided in Fig. 1d–f. These micrographs clearly show the value and change of α_1MEDF_ of untreated 3D Caco-2 luminal cyst models and when treated with either KCN or H_2_O_2_.

### EGF induces a metabolic increase in glucose consumption, lactate production and cell ATP concentration

**Figure 2:**
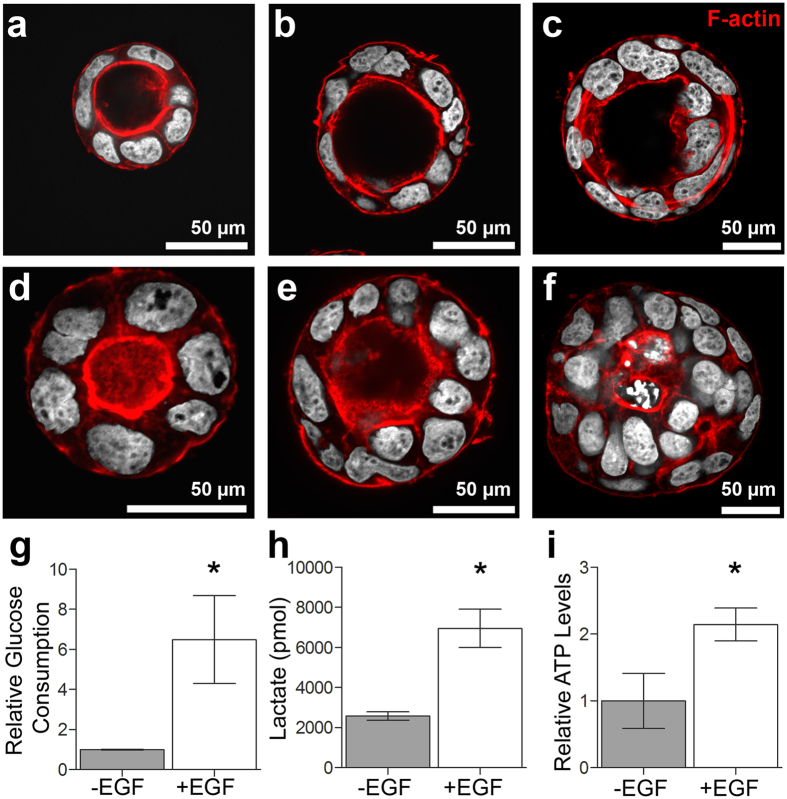
Morphology and metabolic behaviour of 3D Caco-2 luminal cysts treated with EGF. Fluorescence micrographs of 3D Caco-2 luminal cysts: (**a–c**) untreated and (**d–f**) treated with EGF. F-actin is shown in red and nuclei are depicted in white. (**g**) EGF treatment lead to a significant increase in relative glucose consumption (n = 4, *p = 0.0024). (**h**) Lactate production levels in EGF-treated 3D Caco-2 luminal cysts were significantly increased (n = 4, *p = 0.0001). (**i)** A significantly higher content of ATP is seen in EGF-treated 3D Caco-2 luminal cysts (n = 4, *p = 0.0031).

To determine the impact of growth factors on cell metabolic activity, 3D Caco-2 luminal cyst models were left untreated or were treated with EGF and analysed afterwards for their glucose consumption, lactate production and cell ATP concentration (Fig. 2). EGF-treated 3D Caco-2 luminal cyst cultures had a statistically significant increase in glucose consumption compared to untreated models (6.492 ± 2.189 versus 1 ± 0.01663, n = 4, p = 0.0024, Fig. 2g), a statistically significant increase in lactate production (6952 ± 957 pmol versus 2578 ± 206 pmol, n = 4, p = 0.0001, Fig. 2h), and a statistically significant increase in relative cell ATP content (2.145 ± 0.246 versus 1 ± 0.41, n = 4, p = 0.0031, Fig. 2i). 3D Caco-2 models that were untreated or treated with EGF exhibited a typical luminal cyst morphology (Fig. 2a–f).

### Multi-exponential decay fitting can detect a metabolic shift in 3D colorectal cancer models when treated with EGF

**Figure 3:**
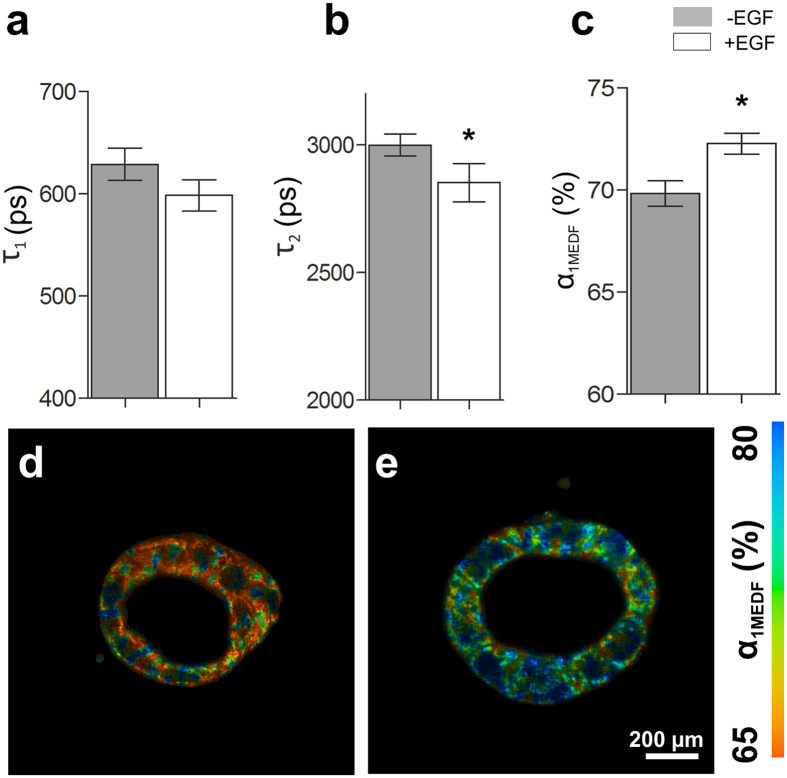
FLIM characteristics of 3D Caco-2 luminal cysts. (**a**) EGF induces a significant decrease in the short fluorescence lifetime *τ*_1_ of NADH (n = 4, *p = 0.002). **(b**) EGF treatment induces a significant decrease in *τ*_2_ (n = 4, *p = 0.0391). (**c**) Treatment of 3D Caco-2 models with EGF induces an increase in the short fluorescence lifetime contribution *α*_1MEDF_ (n = 4, *p = 0.0213). (**d,e**) False color-coded images of (**d**) untreated and (**e**) EGF-treated 3D Caco-2 luminal cyst models indicating the range of *α*_1MEDF_ from 65% (red) to 80% (blue).

3D Caco-2 luminal cysts left untreated or treated with EGF were imaged using FLIM. A bi-exponential decay fitting was applied to the obtained fluorescence decay profiles of the models and from this, the individual contributing lifetime components of free NADH (τ_1_), protein-bound NADH (τ_2_), and the contribution of their lifetimes to the overall signal (α_1MEDF_ being the contribution of τ_1_, Fig. 3) were determined. The treatment of EGF had a statistically significant effect on τ_2_ and α_1MEDF_ in the 3D Caco-2 luminal cyst models (Fig. 3a–c). Specifically, EGF induced a significantly higher value for τ_2_ (2853 ± 75 ps versus 3000 ± 4 ps, n = 4, p=0.0261, Fig. 3b), and a significantly increased value for α_1MEDF_ (72.28 ± 0.52% versus 69.83 ± 0.62%, n = 4, p = 0.002, Fig. 3c). No statistically significant difference in τ_1_ was detected when comparing models treated with EGF with those left untreated. The results from the multi-exponential decay fitting can be viewed qualitatively in the false colour-coded micrographs provided in Fig. 3e,f. These micrographs depict the value and change of α_1MEDF_ of untreated 3D Caco-2 luminal cyst models with and without EGF treatment.

### A custom phasor approach analysis can detect the metabolic shifts of 3D Caco-2 models treated with KCN or H_2_O_2_

**Figure 4:**
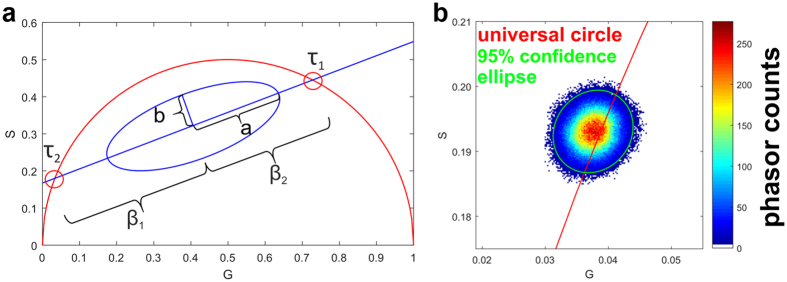
Explanation and validation of phasor plot approach. (**a**) Schematic of phasor plot properties showing the intersections of linear fitted line with universal circle representing lifetimes (e.g. τ_1_ and τ_2_) of contributing fluorophores for bi-exponential decays. Lifetime contributions β_1_ and β_2_ show distance of mean of phasor data from τ_1_ and τ_2_. Shape of the phasor data is determined with calculation of 95% confidence ellipse. Relative lifetime contribution α_i_ (i = 1, 2) is calculated using α_i_ = β_i_/(β_1_ + β_2_). Elliptic ratio *a/b*, determined by semi-major axis *a* and semi-minor axis *b* gives further information about fluorescence properties. (**b**) Phasor plot of FLIM signal from coumarin 6. Green ellipse represents the 95% error ellipse of phasor approach calculations. Ratio between major and minor semi-axis is *a/b* = 1.13 and the subsequent fluorescence lifetime is τ_coumarin_ = 2.48 ns. Calculations were made with harmonic number k = 4 and usage of first quarter of period.

A phasor approach algorithm was developed and implemented as an additional method to interpret the obtained FLIM data. We implemented all phasor methods described in MATLAB (MathWorks Inc., Natick, MA, USA). All source codes, image meta information, and the sample images are publicly available on GitHub (http://doi.org/10.5281/zenodo.159557). This phasor approach enables a discrete plot of the photon distribution and contributing lifetimes (Fig. 4a). For the purpose of validation, data acquired from a FLIM measurement of coumarin 6 was transformed using this phasor approach, and projected to the universal circle (Fig. 4b). This resulted in a fluorescence lifetime calculation of τ = 2.4995 ns, whereby all phasor plot points clustered towards one central point, which is indicative of a mono-exponential decay. This is in agreement with reports of others describing the mono-exponential decay of coumarin 6 with a lifetime of τ = 2.5 ns^31^. This study also investigated the concept of using an elliptical ratio to characterise mono- or bi-exponential decay in FLIM data by reducing all phasor points to the information of the two semi axis of the 95% confidence bounding ellipse. An ellipse with equal semi-axes represents a circle reflecting mono-exponential decay, whereas an elongated ellipse gives evidence of a bi-exponential decay. MEDF requires an assumption of the number of exponential components; however, a phasor approach does not require such initial assumptions.

**Figure 5:**
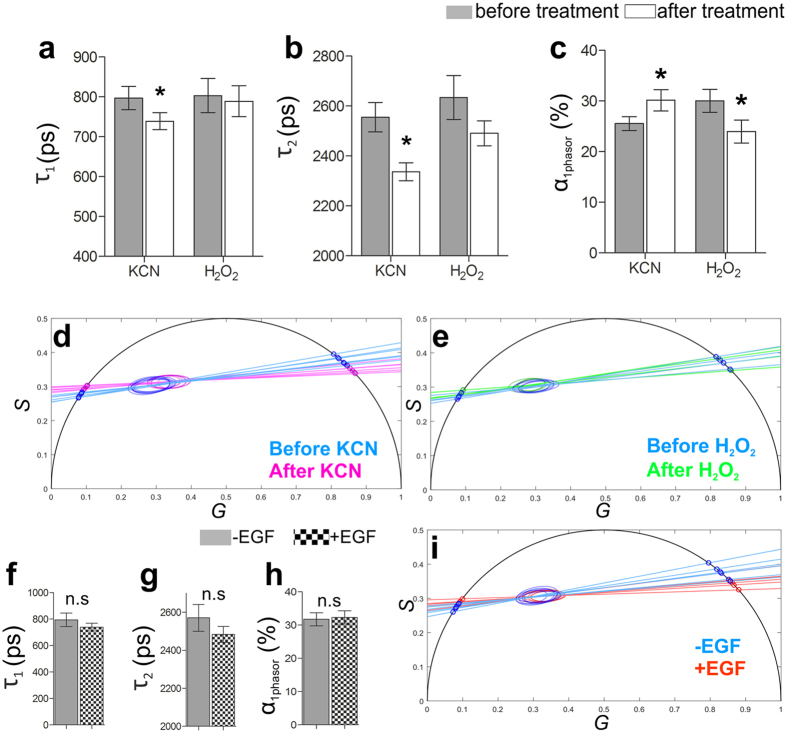
Phasor analysis of FLIM data from KCN-, H_2_O_2_- and EGF-treated 3D Caco-2 models. (**a**) *τ*_1_ derived from phasor analysis before and after treatment with KCN (n = 6) or H_2_O_2_ (n = 4). KCN treatment induces a significant reduction in *τ*_1_ (n = 6, *p = 0.0007). (**b**) KCN treatment of 3D Caco-2 luminal cysts induces a significant decrease of *τ*_2_ (n = 6, *p = 0.0003). (**c**) 3D Caco-2 luminal cysts treated with KCN displayed a significant increase in the short fluorescence lifetime contribution *α*_1phasor_ (n = 6, *p = 0.0169), whereas H_2_O_2_ leads to a decrease in *α*_1phasor_ (n = 4, *p = 0.0113). (**d**) Phasor plot of 3D Caco-2 models before and after treatment with KCN shows 95% confidence ellipses and fitted linear functions on the universal circle. (**e**) Phasor plot of 3D Caco-2 models before and after H_2_O_2_ treatment reveals 95% confidence ellipses and fitted linear functions. (**f–h**) EGF treatment of 3D Caco-2 luminal cysts shows no significant changes in phasor analysis-derived (**f**) short fluorescence lifetime *τ*_1_, (**g**) long fluorescence lifetime *τ*_2_ and (**h**) the short fluorescence lifetime contribution *α*_1phasor_ of NADH (n = 4). (**i**) Phasor plot of untreated and EGF-treated 3D Caco-2 models, showing 95% confidence ellipses and fitted linear functions on the universal circle. All phasor calculations were made with harmonic number k = 2 and usage of first half the period.

3D Caco-2 luminal cysts were imaged using FLIM before and after exposure to KCN or H_2_O_2_. FLIM measurements were then processed using the phasor approach described in this study. In detail, τ_1_, τ_2_ and α_1phasor_ were calculated (Fig. 5a–c) from phasor plots generated using the phasor analysis (Fig. 5d,e). Using this phasor approach, a statistically significant decrease in τ_1_ when treating 3D Caco-2 models with KCN was detected (797 ± 29 ps versus 739 ± 21 ps, n = 6, p = 0.0007). However, treatment of these models with H_2_O_2_ did not elicit a detectable influence on τ_1_ (Fig. 5a). A statistically significant decrease in τ_2_ when treating 3D Caco-2 models with KCN was also detected (2555 ± 59 ps versus 2337 ± 36 ps, n = 6, p = 0.0003). I contrast, treatment with H_2_O_2_ did not elicit a detectable influence on τ_1_ (Fig. 5a). Most importantly, both KCN and H_2_O_2_ were capable of eliciting statistically significant changes in the contribution of the lifetimes τ_1_ and τ_2_ (Fig. 1c). KCN-treated 3D Caco-2 luminal cysts had a significantly higher α_1phasor_ (25.54 ± 1.32% versus 30.16 ± 2.1%, n = 6, p = 0.0169), and H_2_O_2_ caused a statistically significant decrease in α_1phasor_ (30.02 ± 2.26% versus 23.97 ± 2.25%, n = 4, p = 0.0113). These statistically significant shifts were clearly seen in the phasor plots (Fig. 5d,e), whereby the 95% confidence binding ellipse shifted towards the right when 3D Caco-2 models were treated with KCN, and shifted to the left when treated with H_2_O_2_. Applying the phasor analysis to the 3D Caco-2 models treated with EGF did reveal considerable trends that are very similar to the findings of the MEDF approach when compared with the untreated models (Fig. 5f–h, τ_1_, τ_2_ and α_1phasor_), however these trends were not statistically significant. However, when observing the phasor plots of 3D Caco-2 models, untreated or EGF-treated, there was an apparent shift of the 95% confidence bounding ellipses to the right, which is indicative of glycolysis (Fig. 5i).

## Discussion

Intracellular NADH is a universal cofactor that is involved in many metabolic reactions, especially in cell energy metabolism^32^. Changes in intracellular NADH concentration and the ratio of oxidized (NAD+) to reduced (NADH) cofactor are usually associated with a cell transformation^32^,^33^,^34^. In most cancer cells, there is a reduced amount of NADH undergoing oxidation in the mitochondria due to the mutation of enzyme complexes and subsequent uncoupling of the electron transport train^34^. As a compensatory mechanism for ATP production, cancer cells exhibit an elevated glycolytic rate, the “Warburg effect”^35^, leading to larger pools of cytosolic NADH compared to normal cells. Previous studies have shown that the exposure of cell cultures to KCN and H_2_O_2_ can elicit an interruption of respiratory-chain activities (glycolysis versus oxidative phosphorylation) that is detectable using FLIM^12^,^32^,^35^. Here, we described similar effects using a 3D Caco-2 model of colorectal cancer. Blocking cellular respiration with KCN accumulated reduced free NADH within the cells (Fig. 1), and shifted the FLIM phasor distribution toward the free NADH phasor location (Fig. 5d). Inducing oxidative stress within the cells using 100 μM H_2_O_2_ decreased the amount of reduced NADH, thereby decreasing the ratio of free to bound NADH, and shifted the cellular FLIM signature toward the location of the bound NADH (Fig. 5e). We further identified a metabolic trajectory in the phasor plot between a glycolytic phenotype (reducing conditions) and an oxidative phosphorylation phenotype (oxidative conditions) (Fig. 5d and e). We also detected significant changes in the contribution of the lifetimes to the overall FLIM signal via α_1MEDF_, obtained from standard multi-exponential decay fitting of the data (Fig. 1), and α_1phasor_ derived from the custom-generated MatLab approach shared in this study (Fig. 5), which was reflective of a higher free NADH to bound NADH ratio due to treating the cells with KCN. The mean value of the phasor plot got shifted towards τ_1_ from τ_2_. A greater shift towards τ_2_ represented an increase in glycolysis^12^, and the increase in α_1MEDF_ and α_1phasor_ detected in this study validated the MATLAB code presented. This observation is highly significant in that FLIM can be used as a non-invasive characterisation method to elucidate the metabolic kinetics in 3D *in vitro* models; therefore, suggesting a future usability of FLIM to various applications including the screening of pharmaceutical reagents and novel drug candidates on 3D cell-based *in vitro* assays.

Fluorescence decay signatures of NADH have been previously graphically represented on 2D phasor plots, where the decay rates for the pure free or bound species of NADH occupied distinct positions^13^,^14^,^35^. In complex cellular environments, where combinations of free and bound NADH co-exist, fluorescence signatures of decay map experimental points between the extreme phasor plot positions of the pure free and bound NADH reflect the relative ratio of free to bound NADH, and therefore the relative levels of glycolysis and oxidative phosphorylation. Here, we have provided a novel phasor plot approach that can be used on 3D *in vitro* cell culture models, which is available via GitHub (http://doi.org/10.5281/zenodo.159557).

Previous studies have shown that EGF treatment of 3D Caco-2 models increases cellular proliferation and the size of polarized cysts^29^. In this study, we were able to show these effects utilizing both traditional metabolic assays (Fig. 2), and FLIM approaches (Figs 3 and 5). 3D Caco-2 models had a significantly higher consumption of glucose from the supporting media and an increased production of lactate (Fig. 2g,h). Metabolic activity in highly proliferative cells, such as cancer and stem cells, is fundamentally different than that of non-proliferative cells. Warburg first observed that most cancer cells process glucose into lactate regardless of the presence of oxygen^36^. This effect, known as aerobic glycolysis, supports the efficient synthesis of macromolecular components necessary for rapidly dividing cells. Most proliferative cells rely on aerobic glycolysis in contrast to normal differentiating cells that rely primarily on oxidative phosphorylation^35^. 3D Caco-2 models treated with EGF also exhibited a significantly higher content of ATP (Fig. 2i). ATP is efficiently produced during oxidative phosphorylation; as oxidative phosphorylation produces more ATP per molecule of glucose than glycolysis. In light of the data shown, it is expected that a higher production of ATP should be evident when treating 3D Caco-2 models with EGF in line with an increased glucose production. This suggests that the 3D Caco-2 cells are inclined more towards a state of glycolysis rather than one of oxidative phosphorylation. Here, analysis of the FLIM signals of untreated or EGF-treated 3D Caco-2 models using the MEDF approach detected a significant decrease in the lifetime of τ_2_, and a significant increase in α_1MEDF_ (Fig. 3a–c). An increase in α_1_ is indicative of a relative increase of free NADH when compared with protein-bound NADH^11^. This is highly significant as an increase in α_1MEDF_ was also detected when 3D Caco-2 models were treated with KCN to chemically elicit glycolysis. The FLIM data was supported by the vitro cell assays, which strongly suggests that the 3D Caco-2 models treated with EGF are experiencing an increase in glycolysis. Previous studies have similarly reported differences in lifetime contributions for salivary gland stem cells before and after adipogenic differentiation^1^, and different lifetime contributions have been seen in different cancer states of hamster cheek pouches^33^. We then applied a phasor approach towards analysing FLIM measurements acquired from untreated and EGF-treated 3D Caco-2 models (Fig. 5f–i). Although no statistically significant change in τ_1_, τ_2_ or α_1phasor_ was detectable using the phasor approach described in this report, when observing the phasor plot in more detail, it was visible that the data obtained from the EGF-treated 3D Caco-2 models were clearly shifted to the right, which is indicative of glycolysis (Fig. 5i). Indeed, the quantitative outputs of τ_1_, τ_2_ or α_1phasor_ had considerable agreement with the results of the MEDF but were not statistically significant.

To conclude, this study demonstrates that FLIM, in combination with phasor approach analysis, can be used as a valuable tool for the non-invasive and marker-free detection of changes in cell properties within living *in vitro* 3D cell cultures. Both, multi-exponential decay fitting and phasor approach, use different methods to calculate fluorescence lifetimes and their contributions. The application of a phasor plot approach using a custom-generated MATLAB code was capable of detecting the shifts in the decays and contributions of lifetime components of free NADH (τ_1_), protein-bound NADH (τ_2_) achieved using multi-exponential decay fitting. These shifts were statistically significant when treating 3D Caco-2 cultures with KCN and H_2_O_2_. In addition, the custom phasor approach MATLAB code developed in this study was capable of calculating fluorescence lifetimes and their contributions with less initial assumptions than needed when employing the multi-exponential decay fitting, and with less influence of the signal to noise ratio. It also provided a further parameter (such as the elliptical fitting of the phasor coordinates, *a/b*) that cannot be obtained using conventional bi-exponential fitting.

## Material and Methods

### Cell culture and preparation of 3D luminal cyst models

Caco-2 cells were cultured and maintained in RPMI 1640 (Invitrogen^TM^, Karlsruhe, Germany) supplemented with 10% fetal calf serum (FCS) and 1% penicillin streptomycin (P/S) (Invitrogen^TM^) and incubated in a humidified atmosphere of 5% CO_2_ at 37 °C. Upon 70–80% confluency, Caco-2 cells were washed with phosphate buffered saline (PBS) and detached using trypsin (0.05%)/ethylenediaminetetraacetic acid (EDTA). For the generation of 3D luminal cysts, Caco-2 cells were seeded on a bed of growth factor reduced Matrigel^TM^ (BD Biosciences, Heidelberg, Germany) and PureCol^®^-S Collagen (Advanced Biomatrix Inc, San Diego, CA, USA) (1∶1), and overlaid with the standard Caco-2 growth medium containing 2% Matrigel^TM^. After 72 hours of culture, cholera toxin (Sigma Aldrich, Taufkirchen, Germany) was added at a concentration of 100 ng/ml to induce luminal expansion. All imaging was performed after 96 hours of culture whereby the media was exchanged with phenol-free DMEM/F-12 (Thermo Fisher Scientific, Waltham, Massachusetts, USA).

3D Caco-2 luminal cyst models were treated with 4 mM KCN (Sigma Aldrich) dissolved in phenol-free DMEM/F-12 for 1 minute to block mitochondrial respiration and trigger an increase in reduced, free NADH. These models were imaged using FLIM before and again following the addition of KCN. Oxidative stress within 3D models was generated by the addition of 100 μM H_2_O_2_ (Sigma Aldrich), after which amount of reduced NADH decreased, reducing the ratio of free to bound NADH and shifting the cellular FLIM signature towards that of bound NADH.

To elicit the effect of EGF on the cultures, recombinant human EGF (R&D Systems Inc., Abingdon, UK) was added at a final concentration of 10 ng/ml in culture medium from day 0 onwards.

### FLIM imaging

Fluorescence lifetime images of 3D Caco-2 models were acquired with a custom built 5D multiphoton FLIM microscope (JenLab GmbH, Jena, Germany). Two-photon excitation was generated using a Ti: Sapphire femtosecond laser (MaiTai XF1 Spectra Physics, United States, Santa Clara). Fluorescence lifetime signals of NADH were recorded using TCSPC at an excitation wavelength of 710 nm at a laser power of 15 mW. The spectral emission filter for NADH ranged from 425 to 509 nm. FLIM data were recorded at an acquisition time of 180 seconds for 512 × 512 pixels (690 μs/pixel) with 64 time channels. The instrument response function (IRF) was recorded using urea crystal (Sigma-Aldrich) at an excitation wavelength of 920 nm, acquisition time of 60 seconds and with a laser power of 5 mW. FLIM images for validating phasor approach calculations were recorded using coumarin 6 (Sigma-Aldrich, dissolved in 100% ethanol). The solution was imaged at an excitation wavelength of 710 nm, acquisition time of 180 seconds and laser power of 1 mW. The spectral emission filter used for the detection ranged from 425 to 509 nm.

### Multi-exponential decay fitting

Multi-exponential decay fittings (MEDF) were performed using the software SPCImage (Becker & Hickl GmbH, Berlin, Germany). An offset was obtained for every pixel by its lowest photon intensity before excitation. The threshold for calculation was set to 30% of the maximum of photon numbers of the brightest pixel. All calculations were made with two exponential components.

### Phasor approach

Every pixel over a certain threshold level (30% of pixel with highest photon intensity) of the recorded FLIM data set was transformed using Fourier transform to generate G-S-plane phasor coordinates using a custom-generated code written in MATLAB (The MathWorks Inc., Massachusetts, USA). All source codes, image meta information and the sample images are publicly available on GitHub (http://doi.org/10.5281/zenodo.159557). As demonstrated in equation (5) together with the TLS fit, the contribution lifetimes of the 3D models were calculated. By orthogonal projection of all data points (that are not present on the fitted line - the line connecting (τ_1_ and τ_2_)), a distribution of phasor points between τ_2_ and τ_1_ were calculated. α_1phasor_ is hereby not directly comparable to α_1MEDF_, due to the non-linear mathematical behaviour of data in the phasor plane, but it gives a variable to distinguish between different phasor plots. While α_1,MEDF_ is linear to the relative photon contribution of the fluorophore with the fast fluorescence lifetime, α_1phasor_ is more complicated due to mathematical distortions caused by the Fourier transformation.

For all data points plotted on the G-S phasor plane, a 95% confidence ellipse was calculated and applied with a long half axis *a* and short half axis *b* as additional parameters for the analysis of the phasor plot (Fig. 4a) This relation of *a/b* serves as a metric for the ellipse form. A minimum ellipse ratio *a/b* = 1 infers that the ellipse is a circle. The higher the ratio, the longer and thinner the ellipse becomes. A higher *a/b* the probability increases justifies the assumption of a bi-exponential decay and the deviation of lifetime contribution α_1phasor_.

### Glucose, lactate and ATP assays

Glucose and lactate measurements were performed according to the manufacturer’s guidelines on media collected from 3D Caco-2 luminal cyst cultures at day 4 of culture using the Glucose (GO) Assay Kit (GAGO-20, Sigma Aldrich) and Lactate Assay Kit (MAK064, Sigma Aldrich). ATP measurements were performed according to the ENLITEN ATP Assay System (#FF2000, Promega Corporation, Madison, Wisconsin, USA).

To quantify cell numbers in the 3D models, the content of total DNA in the samples was measured using the Quant-iT™ Picogreeen^®^ dsDNA Assay Kit (Invitrogen). Samples were briefly washed with PBS and incubated at 60 °C in Proteinase K for 12 hours, after which the DNA content was assessed according to the manufacturer’s guidelines. All measurements were performed in triplicates.

### Fluorescence microscopy

3D Caco-2 models were washed twice with PBS and fixed using 4% paraformaldehyde (Sigma-Aldrich) for 15 minutes. The samples were then permeabilised using a 0.1% Triton-X-100 solution made in PBS for 15 minutes and stained for F-actin using phalloidin for 30 minutes (Alexa Fluor^®^ 594 Phalloidin, Invitrogen) and cell nuclei using a 4’,6-diamidino-2-phenylindole (DAPI, Sigma-Aldrich) solution for 10 minutes. Mounting was performed using ProLong^®^ Gold antifade mounting medium (Molecular Probes, life technologies, Germany). Fluorescence images were acquired using a LSM 710 system or a AxioObserver.Z1 (Carl Zeiss, Jena, Germany).

### Statistical analysis

Results are depicted throughout this manuscript as the mean ± standard deviation. Statistical analysis was performed using PRISM (GraphPad Software, La Jolla, CA, USA). All data sets were assumed to be normally distributed which was confirmed using the Shapiro-Wilks test to test for normal distribution. Student’s t-test (two-tailed, unpaired) was performed where appropriate to determine statistically significant differences between two groups. In experiments where FLIM measurements were performed before and after the addition of KCN or H_2_O_2_, Student’s t-test (two-tailed, paired) was applied. Statistical significance was set at p < 0.05.

## Additional Information

**How to cite this article:** Lakner, P. H. *et al*. Applying phasor approach analysis of multiphoton FLIM measurements to probe the metabolic activity of three-dimensional *in vitro* cell culture models. *Sci. Rep.* **7**, 42730; doi: 10.1038/srep42730 (2017).

**Publisher's note:** Springer Nature remains neutral with regard to jurisdictional claims in published maps and institutional affiliations.

## Supplementary Material

Supplementary Data
