# Supplementary material for: Applying phasor approach analysis of multiphoton FLIM measurements to probe the metabolic activity of three-dimensional in vitro cell culture models
Source: Sci Rep. 2017 Feb 13;7:42730. doi: 10.1038/srep42730 (PMC5304149; doi:10.1038/srep42730)
Supplement: Supplementary Data [file srep42730-s1.pdf]

Applying phasor approach analysis of multiphoton FLIM measurements to probe the metabolic activity of three-dimensional in vitro cell culture models

Pirmin H. Lakner[a#], Michael G. Monaghan[a,b,c,d#], Yvonne Möller[e,f], Monilola A. Olayioye[e,g], Katja Schenke-Layland[a,d,h,*]

[a] Department of Women's Health, Research Institute for Women's Health, University Hospital of the Eberhard Karls University Tübingen, Tübingen, Germany

[b] Trinity Centre for Bioengineering, Trinity Biomedical Sciences Institute, Trinity College Dublin, Dublin, Ireland

[c] Department of Mechanical and Manufacturing Engineering, School of Engineering, Trinity College Dublin, Dublin, Ireland

[d] Department of Cell and Tissue Engineering, Fraunhofer Institute for Interfacial Engineering and Biotechnology (IGB), Stuttgart, Germany

[e] Institute of Cell Biology and Immunology, University of Stuttgart, Stuttgart, Germany

[f] Center for Personalised Medicine (ZPM), University Hospital of the Eberhard Karls University Tübingen, Tübingen, Germany

[g] Stuttgart Research Center Systems Biology, University of Stuttgart, Stuttgart, Germany

[h] Department of Medicine/Cardiology, University of California Los Angeles (UCLA), Los Angeles/CA, USA

# Authors contributed equally

Multi-exponential decay fitting

Fluorescence systems with N different fluorescence lifetimes emit exponentially decaying fluorescence intensities:

$$F(t \geq 0) = \sum_{n=1}^{N} \gamma_n \exp(-\frac{t}{\tau_n})$$

with fluorescence lifetimes τ_n and their contributions γ_n.

The relative contribution of the fluorescence lifetime is calculated using:

$$\alpha_i = \frac{\gamma_i}{\sum_{n=1}^{N} \gamma_n}$$

In a perfect system, a fluorophore is excited with a Dirac-shaped laser pulse at time $\mathrm{t} = 0$. Therefore, its initial intensity is:

$$F(t < 0) = 0$$

Due to instrumental effects, photon arrival times at photon detection devices can be variable and delayed. This effect fluctuates between devices and is also responsive to changes in time and environmental settings. It is called the instrument response function (IRF). The recorded signal $\mathrm{I(t)}$ from a fluorescence system is the convolution of its fluorescence intensity $\mathrm{F}\ (\mathrm{t})$ and the IRF ($\mathrm{R(t)}$)[1]:

$$I(t) = F(t) \otimes R(t)$$

Other effects that influence recorded fluorescence intensities include signals from backscattering or second harmonic generation ($\mathrm{S(t)}$) and offset effects (C), which can be caused by ambient light, noise from the detector, and also after-pulses due to feedback from

the detector. By taking these effects into account, the equation for recorded photon intensity $\tilde{I}$(t) expands to:

$$\tilde{I}(t) = I(t) + S(t) + C$$

With multi-exponential decay fitting, this function can be applied to the measured data set using several iterative steps[2]. In order to gain relevant results, limiting parameters must be defined, such as the minimum and maximum limits for lifetimes or the number of exponential decay components. The number of iterative steps is limited to enable the analysis within an acceptable time frame.

Phasor plot analysis

Fluorescence lifetimes can also be calculated using a phasor approach, first described by Jameson et al. in 1984[3]. It applies Fourier transform to an exponential decay and enables an identification of the decay components. For simplification, a basic form of an exponential decay considered whereby such a fluorescent system with lifetime τ is given by

$$F(t) = \begin{cases} \exp\left(-\frac{t}{\tau}\right), & \text{for } t \geq 0 \\ 0, & \text{for } t < 0 \end{cases}$$

No signal is assumed before $t = 0$, and this function can be transformed to an application of a Fourier transform. Normally, a single fluorescence decay is not periodic; however, by excitation of fluorescence using a pulsed laser, exponential decay can be repeated periodically at a laser repetition frequency f and a period length of:

$$T = \frac{1}{f}$$

Applying Fourier transform on the given exponential decay gives:

$$\hat{f}(\omega) = \int_{-\infty}^{\infty} f(t) \exp(-ik\omega t)\, dt$$

$$= \int_{-\infty}^{0} f(t)\exp(-ik\omega t)\,dt + \int_{0}^{\infty} f(t)\exp(-ik\omega t)\,dt$$

$$= 0 + \int_{0}^{\infty} f(t)\exp(-ik\omega t)\,dt$$

where $\omega = 2\pi f$ is the angular frequency of laser repetition and k is the harmonic number. Incorporating this into the equation for $F(t)$, the integral for the negative regime becomes 0. Due to the presence of periodic behaviour, the integration interval in the positive regime can be reduced to the first period. All data after the end of the first period gets projected into the first period by all (virtual) decays before the considered period. Therefore, every period contains all data of a whole exponential decay. This changes the initial elementary exponential decay to:

$$F(t) = \sum_{n=0}^{\infty} \exp\left(-\frac{t + n \cdot T}{\tau}\right)$$

with $t \in [0,T]$. Thereafter the equation for Fourier transform reduces to:

$$\hat{f}(\omega) = \int_{0}^{T} F(t)\exp(-ik\omega t)\,dt$$

which results in a generation of a complex number. With Euler's formula:

$$\exp(iz) = \cos(z) + i\sin(z)$$

the complex number can be divided into its real and imaginary component. This generates the following equations:

$$G_{m,n} = Re(\hat{f}) = \frac{\int_0^{\infty} F_{m,n}(t)\cos(k\omega t)\,dt}{\int_0^{\infty} F_{m,n}(t)dt}$$

$$S_{m,n} = Im(\hat{f}) = \frac{\int_0^{\infty} F_{m,n}(t)\sin(k\omega t)\,dt}{\int_0^{\infty} F_{m,n}(t)dt}$$

where $F(t)$ is the photon intensity at a row m and column n in a recorded matrix of FLIM data and k the harmonic number of the Fourier transform. Both integrals are divided by the intensity of the signal for normalization. For mono-exponential decays these equations can be written as:

$$G_{m,n} = Re(\hat{f}) = \frac{1}{1+(k\omega\tau)^2}$$

$$S_{m,n} = Im(\hat{f}) = \frac{k\omega\tau}{1+(k\omega\tau)^2}$$

With these two equations, it is possible to calculate the lifetime for the measured decay[4]:

$$\tau = \frac{1}{k\omega}\frac{S}{G}$$

By plotting the imaginary versus the real component of the transform, lifetimes of emitted photons within pixels of an image, obtained using FLIM, are assigned to a point in a phasor plot. For mono-exponential decay all points lay on a semicircle beginning at coordinate (1.0) and moving counter clockwise to (0.0) on a semicircle with radius 0.5 [5,6], which is commonly known as the 'universal circle' in literature[7]. Lifetimes of mono-exponential decays can be calculated directly by the position on the semicircle using the equation above. Multi-exponential decays give phasor points/ clusters within the semicircle. FLIM images with multi-exponential decays yield clouds of phasor data points within the semicircle.
The main advantage of the phasor approach, when compared to MEDF, is that it requires less initial assumptions and no iterative calculations[8].

References

1. Warren, S.C. et al. Rapid global fitting of large fluorescence lifetime imaging microscopy datasets. *PLoS ONE* **8**, e70687 (2013).
2. Becker, W. et al. Fluorescence lifetime imaging by time-correlated single-photon counting. *Microsc Res Tech* **63**, 58-66 (2004).
3. Jameson, D.M., Gratton, E. & Hall, R.D. The measurement and analysis of heterogeneous emissions by multifrequency phase and modulation fluorometry. *Appl Spectrosc Rev* **20**, 55-106 (1984).
4. Leray, A. et al. Quantitative comparison of polar approach versus fitting method in time domain FLIM image analysis. *Cytometry A* **79**, 149-58 (2011).
5. Fereidouni, F., Esposito, A., Blab, G.A. & Gerritsen, H.C. A modified phasor approach for analyzing time-gated fluorescence lifetime images. *J Microsc* **244**, 248-58 (2011).
6. Grecco, H.E., Roda-Navarro, P. & Verveer, P.J. Global analysis of time correlated single photon counting FRET-FLIM data. *Opt Express* **17**, 6493-508 (2009).
7. Digman, M.A., Caiolfa, V.R., Zamai, M. & Gratton, E. The phasor approach to fluorescence lifetime imaging analysis. *Biophys J* **94**, L14-L16.
8. Stefl, M., James, N.G., Ross, J.A. & Jameson, D.M. Applications of phasors to in vitro time-resolved fluorescence measurements. *Anal Biochem* **410**, 62-9 (2011).
